# The impact of near visual impairment on instrumental activities of daily living among community-dwelling older adults in Selangor

**DOI:** 10.1186/s13104-021-05813-3

**Published:** 2021-10-24

**Authors:** Qiu Ting Kee, Mohd Harimi Abd Rahman, Norliza Mohamad Fadzil, Zainora Mohammed, Suzana Shahar

**Affiliations:** 1 Optometry and Vision Sciences Programme, Center for Rehabilitation and Special Needs Studies, Faculty of Health Sciences, Universiti Kebangsaan Malaysia, Wilayah Persekutuan , Kuala Lumpur Malaysia; 2 Dietetic Programme, Center of Healthy Ageing and Wellness, Faculty of Health Sciences, Universiti Kebangsaan Malaysia, Wilayah Persekutuan, Kuala Lumpur Malaysia

**Keywords:** Aging, Contrast sensitivity, Visual acuity, Visually impaired, Quality of life

## Abstract

**Objective:**

Near visual impairment (VI) is a common disability in an aging population. Near vision is crucial in activity of daily living including reading, smartphone and computer use and meal preparation. This study was conducted to determine the association between near visual acuity (VA) and contrast sensitivity (CS) with activity of daily living (ADL) among visually impaired older adults.

**Results:**

A total of 208 participants aged  ≥  60 were recruited from the population-based longitudinal study on neuroprotective model for healthy longevity. Habitual near VA and CS were measured using Lighthouse near VA chart and Pelli-Robson CS chart, respectively. Lawton instrumental activities of daily living (IADL) was used to assess ADL. There are 41.8% participants with near visual impairment and 28.7% among them had IADL disability. Independent t test showed significant lower mean IADL score among visually impaired participants [t(206)  =  2.03, p  =  0.04]. IADL score significantly correlated with near VA (r  =   − 0.21, p  =  0.05) but not with CS (r  =   − 0.14, p = 0.21). Near VA (B  =   − 0.44, p  =  0.03) and age (B  =   − 0.07, p  =  0.01) significantly predicted IADL. The findings show poorer VA renders higher IADL disability, which may necessitate interventions to improve ADL among visually impaired older adults.

## Introduction

Visual impairment (VI) is a common disability among older adults worldwide and its prevalence increases with advancing age [1]. Globally, the prevalence of VI reported was 7.7% in which 64.2% from it was among population aged  ≥  50 and 419 million older adults had near VI due to uncorrected presbyopia [2]. Most of the studies focus on distance VI and often ignore near VI as an important aspect of visual disability [3–7]. Good near vision is crucial in daily activities including reading, digital devices usage and preparing meal [8].

Activity of daily living (ADL) refers to the fundamental skills necessary for daily self-care which further categorized into basic ADL (BADL) and instrumental ADL (IADL) [9]. BADL is functional skills that are mastered early in life, including feeding, personal hygiene, dressing, ambulating, continence, and toileting. IADL involves more complex thinking and organizational skills such as housekeeping, managing finances, handling medications and meal preparation [10]. Significant association between VI and IADL limitation but not ADL limitation was found as ADL involved automatic tasks that learned through repetitive practices, requiring less cognitive and visual skills [11, 12]. A high prevalence of IADL limitation among Malaysian older adults (42.5–58.1%) was reported but the effect of poor near vision on IADL remain unclear as vision was not assessed [13–15].

VI led to person-environment misfit, causing difficulty in handling daily tasks even at familiar environment [16]. There were 18.9% older adults with near VI having IADL limitation and a higher prevalence (27.6%) among those receiving home care in Ontario, Canada [17, 18]. Ishihara et al. [19] suggested the importance of contrast sensitivity (CS) in handling of small things (e.g., coins, telephone, medicine), perception of step edges and detection of obstacles among elderly. Hence, further investigation on the relationship between CS and IADL was suggested [11, 18, 20]. Currently, evidence on the association between near visual acuity (VA) and CS with IADL among community-dwelling older adults in Malaysia is still lacking. Research conducted overseas may not be applicable to the Malaysian context as IADL can be influenced by environmental, societal, and cultural factors [12, 13, 18, 21]. As the Malaysian elderly population is expanding following improved healthcare promoting longevity [7, 22, 23], it was estimated that VI and physical disability will increase concurrently. We hypothesized that near VA and CS are associated with IADL score among older adults. Hence, this study was conducted to determine the association between near VA and CS with IADL among visually impaired older adults in Selangor, Malaysia.

## Main text

### Methods

Participants were recruited from 12 places randomly selected from Selangor state (Kuala Langat, Kajang, Tanjung Sepat, Sungai Pelek, Tanjung Karang, Kuala Selangor, Sekinchan, Keramat, Klang, Petaling Jaya, Kelana Jaya and Batu 9 Cheras) from August 2018 to May 2019 and were analyzed cross-sectionally. Sample size, n_0_ was calculated based on Cochran formula [25]:

$${\text{n}}_{{0}} {\text{ = Z}}^{{2}} {\text{pq/e}}^{{2}}$$

where Z^2^ is 1.96 for 95% confidence interval, p, estimated proportion is 2.04% IADL limitation among VI population [18], q is 1 − p and e, precision level is 0.05. The total sample size required was 40 after added with 20% drop out.

Inclusion criteria were older adults aged  ≥  60 and without documented major psychiatric illnesses or mental disorders. Those with Mini-Mental State Examination (MMSE) score  ≤  14, indicating moderately severe or severe cognitive impairment were excluded [26]. This study adhered to the Declaration of Helsinki and was approved by the Medical Research and Ethics Committee of Universiti Kebangsaan Malaysia (UKM1.21.3/244/NN-2018-145).

A total of 230 participants agreed to participate and signed informed consent was obtained. After excluding 22 participants due to missing data or MMSE score  ≤  14, the sample size of 208 participants remained. Participants were interviewed on demographic information including age, gender, races, and educational level.

### Assessment

Habitual near VA was measured monocularly at 40 cm using Lighthouse near VA chart (Precision Vision, USA). The smallest lines of the chart that participants able to read was recorded in M unit and  ≤  0.8 M was defined as no VI whereas  >  0.8 M as VI [27]. The lower M score in Lighthouse near chart indicates better near VA.

CS was measured binocularly using Pelli-Robson Contrast Sensitivity chart at 1 m with chart luminance of 85 cd/m^2^ [28]. The lowest triplet of letters with at least two of the three letters read correctly was recorded as log CS.

Malay version Lawton IADL was administered to assess independent living skills [29]. IADL questionnaire assessed for seven items including phone usage, shopping, doing housework, finance management, traveling, food preparation and taking medications [24]. Each item was scored as 0 (not able/dependent to perform task), 1 (perform task with assistance) or 2 (perform task independently). The total IADL score is 14, in which lower IADL score showed severe IADL disability was defined as assistance needed for the task or not able to do the task at all [30].

Malay version of MMSE for visually impaired (M-MMSE-blind) was used to assess cognitive function [31]. The score for M-MMSE-blind was calculated by eliminating items involving vision (naming, performing a three-stage command, following a written instruction, writing a sentence, and copying), leaving a total score of 22.

### Statistical analysis

All statistical analyses were conducted through IBM SPSS Statistics for Windows, version 23.0 (IBM Corp., Armonk, N.Y., USA). Descriptive statistics was used to present the mean and standard deviation for continuous data whereas frequency and percentage for categorical data. The data was normally distributed (p  >  0.05). Mean IADL score between VI and non-VI groups were compared using independent t test. Correlations between near VA and CS with IADL score was determined with Pearson correlation. An entry criterion of p  <  0.20 was used in simple linear regression to determine the association between near VA and CS with IADL and significance was determined at p  <  0.05 level [32].

## Results

From a total of 208 participants with mean age of 72.39  ±  5.33, 41.8% had near VI (Table 1). Among VI participants, female (63.2%) slightly outnumbered male (36.8%) and majority are Chinese (70.1%). Most of them received primary (34.5%) and secondary education (39.1%). The results also show that most of the participants have more than one health problem. About one third with diabetes mellitus (34.5%) and osteoarthritis (32.2%) whereas about half with hypertension (52.9%) and high cholesterol (51.7%).

**Table 1:** Characteristic and clinical assessments of all participants according to VI status

Variables	Total (n = 208)	No VI (n = 121)	Near VI (n = 87)
Age, mean ± SD (range)	72.39 ± 5.33 (64–88)	71.44 ± 5.00 (65–86)	73.72 ± 5.52 (64–88)
Gender, n (%)			
Male	90 (43.3)	58 (47.9)	32 (36.8)
Female	118 (56.7)	63 (52.1)	55 (63.2)
Races, n (%)			
Malay	72 (34.6)	56 (46.3)	16 (18.4)
Chinese	106 (51.0)	45 (37.2)	61 (70.1)
Indian	30 (14.4)	20 (16.5)	10 (11.5)
Educational level, n (%)			
No formal education	26 (12.5)	10 (8.3)	16 (18.4)
Primary education	61 (29.3)	31 (25.6)	30 (34.5)
Secondary education	90 (43.3)	56 (46.3)	34 (39.1)
Tertiary education	31 (14.9)	24 (19.8)	8 (8.0)
Health status, n (%)			
Diabetes mellitus	68 (32.7)	38 (31.4)	30 (34.5)
Hypertension	110 (52.9)	64 (52.9)	46 (52.9)
High cholesterol	116 (55.8)	71 (58.7)	45 (51.7)
Osteoarthritis	62 (29.8)	34 (28.1)	28 (32.2)
Hip fracture	7 (3.4)	4 (3.3)	3 (3.4)
Near VA (M), mean ± SD (range)	0.95 ± 0.59 (0.30–5.00 M)	0.60 ± 0.15 (0.30–0.80 M)	1.42 ± 0.65 (1.00 – 5.00 M)
CS (log CS), mean ± SD (range)	1.53 ± 0.18 (0.75–1.95 log CS)	1.59 ± 0.12 (1.05–1.95 log CS)	1.44 ± 0.21 (0.75–1.80 log CS)
M-MMSE-blind score, mean ± SD (range)	18.70 ± 2.93 (9–22)	19.21 ± 2.64 (10–22)	17.99 ± 3.16 (9–22)
IADL score, mean ± SD (range)	13.55 ± 1.08 (7–14)	13.69 ± 0.85 (9–14)	13.36 ± 1.32 (7–14)

*VI* visual impairment; *N* number; *SD* standard deviation

The mean of near VA, CS, M-MMSE-blind score and IADL score among VI participants were 1.42  ±  0.65 M, 1.44  ±  0.21 Log CS, 17.99  ±  3.16 and 13.36  ±  1.32, respectively. Among the participants with near VI, 28.7% (n  =  25) had IADL disability, and 71.3% (n  =  62) had no IADL disability. For participants without near VI, 17.4% (n  =  21) had IADL disability, and 82.6% (n  =  100) had no IADL disability. Independent t test showed significant lower IADL score in VI group as compared to non-VI group [t(206)  =  2.03, p  =  0.04].

Among VI group, independent t-test revealed no significant different in mean IADL score among gender [t(85)  =  0.75, p  =  0.46] (Table 2). ANOVA test showed no significant different in mean IADL score among different races [F(2,84)  =  0.12, p  =  0.89] and educational level [F(3,83)  =  2.15, p  =  0.10].

**Table 2:** IADL score stratified by gender, races, and educational level among VI participants (n  =  87)

Variables	IADL score			
	Mean	SD	Range	p
Gender				
Male	13.47	1.30	9–14	0.46^a^
Female	13.29	1.34	7–14	
Races				
Malay	13.44	0.81	12–14	0.91^b^
Chinese	13.31	1.44	7–14	
Indian	13.50	1.27	10–14	
Educational level				
No formal education	13.25	1.84	7–14	0.10^b^
Primary education	12.93	1.55	9–14	
Secondary education	13.68	0.73	11–14	
Tertiary education	13.86	0.38	13–14	

*IADL* Lawton Instrumental Activities of Daily Living; *VI* visual impairment; *N* number; *SD* standard deviation

^a^Independent t test

^b^One-way ANOVA

Pearson correlation showed higher IADL score (less disability) significantly correlated with better near VA (lower M score in Lighthouse near chart) (r =  − 0.21, p  =  0.05), but not with CS (r  =  − 0.14, p = 0.21) (Table 3).

**Table 3:** Pearson correlation between age, cognition and visual function with IADL among VI participants (n  =  87)

	Age	M-MMSE-blind score	Near VA	CS	IADL score
Age	1.00				
M-MMSE-blind score	− 0.18*	1.00			
Near VA	− 0.07	− 0.02	1.00		
CS	− 0.14*	0.02	0.54**	1.00	
IADL	− 0.30**	0.21*	− 0.21*	− 0.14	1.00

*IADL* Lawton Instrumental Activities of Daily Living; *VA* visual acuity; *CS* contrast sensitivity; *M-MMSE-blind* Malay version of Mini-Mental State Examination for visually impaired; *VI* visual impairment; *N* number

^*^p  <  0.20

^**^p  <  0.05

A multiple linear regression was conducted to predict IADL score based on near VA, age and M-MMSE-blind score among VI group. A significant regression equation was found [F(3,83)  =  5.37, p  <  0.01], with R^2^ of 0.16. Participants’ predicted IADL score is equal to 17.83 − 0.44(NEAR VA, in M unit) − 0.07(AGE, in year)  +  0.06(M-MMSE-BLIND SCORE, in point). Participants’ IADL score decreased by 0.44 M unit for each of near VA, 0.07 year for each of age and 0.06 point for each of M-MMSE-blind score. Better near VA (B  =  − 0.44, p  =  0.03) and increasing age (B  =  − 0.07, p  =  0.01) were significant predictors of IADL score whereas M-MMSE-blind (p  =  0.14) did not.

## Discussion

To our knowledge, this is the first study on the association between near VA and IADL among visually impaired older adults in Malaysia. The present study, conducted among community-dwelling older adults with VI, highlights a significant inverse relationship between near VA with IADL. The poorer the near VA, the more severe the IADL disability among older adults with VI. Previous studies found that self-reported poor near vision doubled the risk of IADL limitation [OR  =  2.10, 95% CI(1.52, 2.90)] [12] and the risk increased with severity of VI, from 2.2 times in mild near VI to 3.6 times in moderate to severe near VI [21]. A more recent study reported a significant association between near VI (Parinaud score  >  2) and IADL limitation both cross-sectionally [OR  =  1.60, 95% CI(1.2, 2.0)] and longitudinally [OR  =  1.2, 95% CI(1.0, 1.4)] [18]. In addition, near VI causes greater risk of developing IADL limitation in all tasks except shopping and phone usage. However, variations in near VA assessment and definitions on VI and IADL limitation across these studies limit direct comparison of the findings.

The findings of this study support previous study in which elderly with self-reported fair to poor vision experienced difficulty in any IADL activity, especially meals preparation, phone usage and money management [33]. Older adults with VI experienced reading-related barriers in all activities in IADL such as reading expiration dates, medication instructions, product labels and prices, identifying buttons on appliances and dealing with coins or bills [34]. All the tasks as previously mentioned required good visual abilities as suggested by Berger and Porell [12]. They stressed on the necessity of visual skills, fine motor dexterity, and cognitive skills especially in phone use, medication, and finance management.

Rubin et al. [35] found that CS was a significant risk factor for IADL limitation among elderly [OR  =  1.93, 95% CI(1.30, 2.87)] but the association did not persist after adjustment for age, gender, race and chronic medical conditions [OR  =  1.45, 95% CI(0.95, 2.22)] [35]. However, this study did not find any significant correlation between CS and IADL score. This may be because the mean CS (1.53  ±  0.84 log units) in this study was above the level of CS that can cause disability. West et al. [36] reported that mobility and heavily visual intensive tasks were affected when CS are worse than 0.9 log units and 1.4 log units, respectively [36].

The association between various subitems in MMSE with IADL have been commonly reported [37–39]. For instance, “orientation to time” in MMSE was reported to be associated with “ability to handle finances” and “responsibility for own medications” in IADL [40]. Similar to study by Safak et al. [39], our findings show significant correlation between MMSE and IADL. However, different from study by Lee et al. and Han et al. [37, 38], there is no significant association in regression analysis. Discrepancy in findings was likely due to our study having included only older adults with no measurable cognitive impairment, whereas others included those with mild cognitive impairment, dementia, and Alzheimer’s Disease.

Our study suggests that age was a significant predictor of IADL score, which agrees with previous findings [13, 41]. Aging is associated with generalized deterioration of body organs and systems, lowering effectiveness of physiological functions, which lead to a greater risk for various chronic disease and inadaptability among elderly. Another study reported an opposite trend, in which individuals with earlier onset of VI have less IADL disability as they may be equipped with skills to overcome the disability compared to those with onset of VI at older age [42].

The strengths of this study include objective measurement of near VA and classification of near VI based on ICD-11 for Mortality and Morbidity Statistics [27]. Secondly, M-MMSE-blind was used in analysis as controlling factors thus addressing the potential influence of cognitive impairment among VI subjects.

This study suggested a significant inverse association between near VA and IADL. There may be a need for interventions appropriate to the visually impaired such as optical correction and low vision rehab, to optimize IADL and mitigate disability. Concomitant public health measures may include activities to improve public awareness regarding the availability of such resources and programs in the community.

## Limitations

This study did not use a specific ADL questionnaire for VI population. Further study should be conducted using a specific ADL questionnaire designed for VI population in order to gain a more specific or focused finding. As this study was only conducted in a Malaysian context, its findings might only be relevant to the Malaysian older adult’s population. It is interesting to know if same findings could be found in other countries or cultural settings.

## Data Availability

The author confirm that all relevant data are included in the article.

## Abbreviations

VI: Visual impairment
ADL: Activity of daily living
BADL: Basic activity of daily living
IADL: Lawton instrumental activities of daily living
CS: Contrast sensitivity
VA: Visual acuity
M-MMSE-blind: Malay version of Mini-Mental State Examination for visually impaired
n: Number
SD: Standard deviation
